# Supplementary figures and images for: Leishmania amazonensis promastigotes in 3D Collagen I culture: an in vitro physiological environment for the study of extracellular matrix and host cell interactions
Source: PeerJ. 2014 Apr 3;2:e317. doi: 10.7717/peerj.317 (PMC3994643; doi:10.7717/peerj.317)

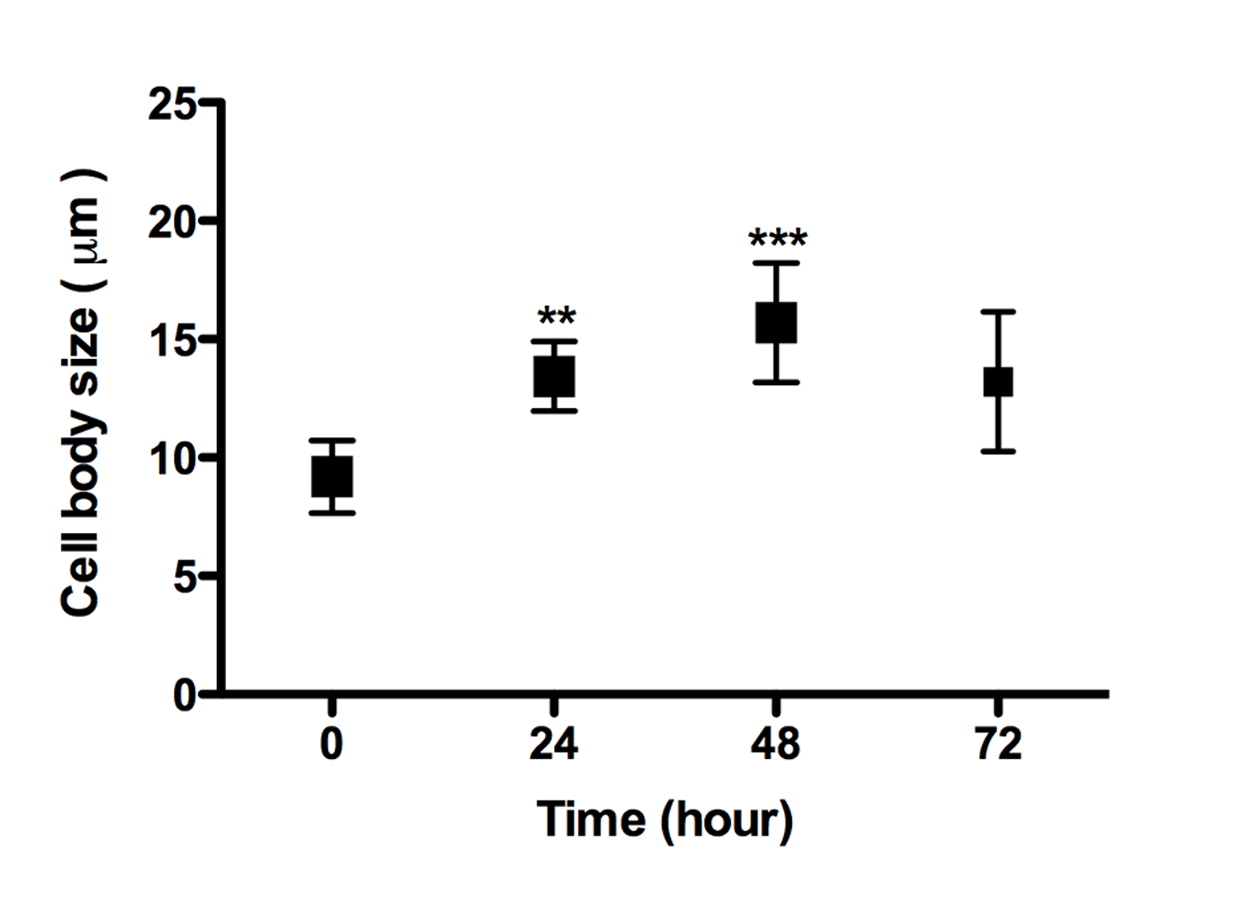

Supplement: Supplemental Information 1 — Promastigote cell body size over cultivation time inside the 3D COL I matrix. Whiskers represent the standard deviation. 1-way ANOVA **, p < 0.001; ***, p < 0.0001; n = 15. [file peerj-02-317-s001.png]

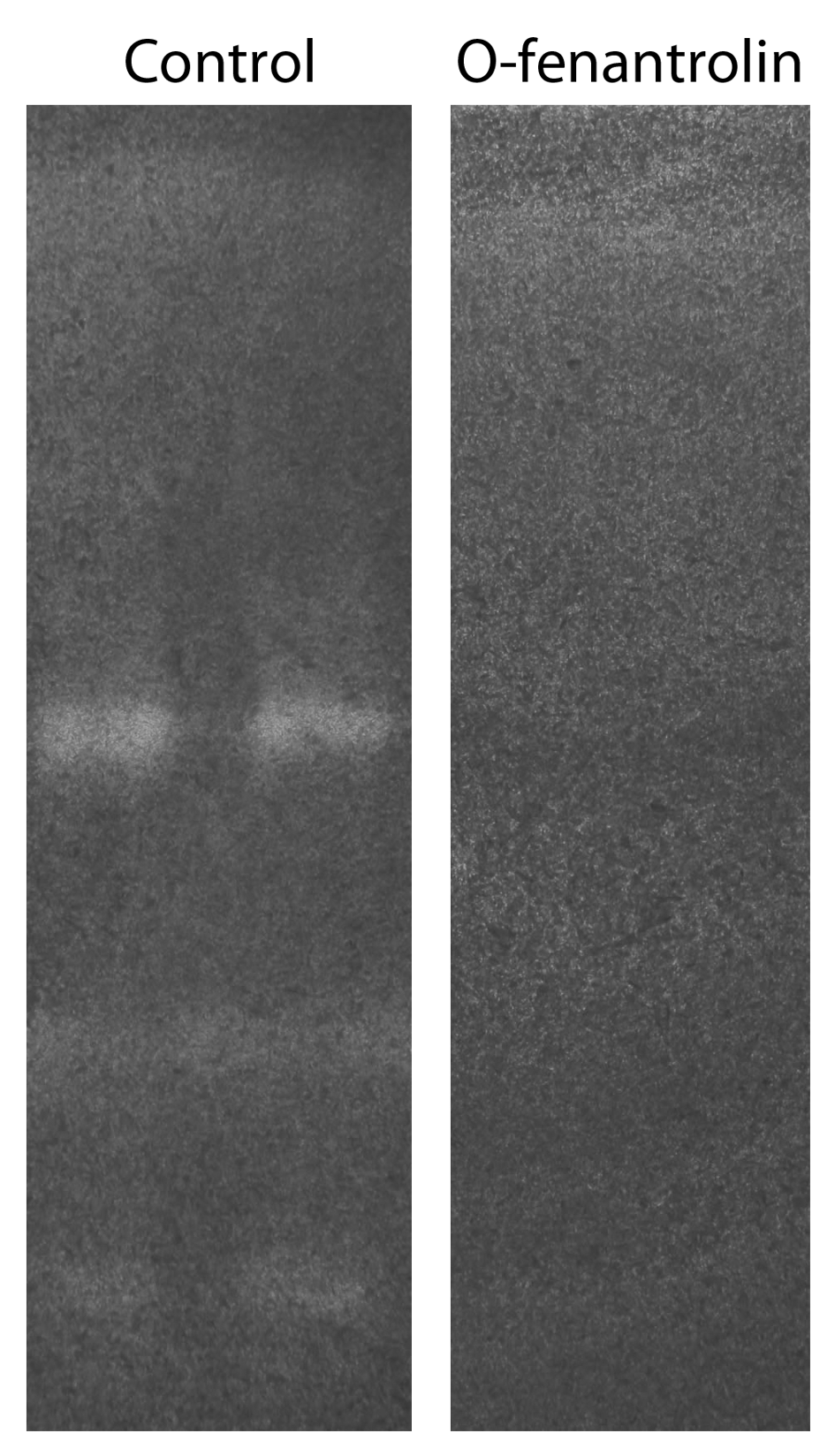

Supplement: Supplemental Information 2 — Scanned pictures of COL I zymography gels. Gels containing two different samples of 72 h 3D COL I matrix promastigote cultivation supernatant. After electrophoresis the gel was cut in three parts and each part was separately incubated with 50 mM Tris, 10 mM CaCl2, 1 mM DTT buffer (pH 6.8) for 48 h with or without (control) O-phenantroline (5 mM). O-phenantroline presence in the buffer completely inhibited the COL I protease relative band. [file peerj-02-317-s002.png]
