# Supplementary material for: Leishmania amazonensis promastigotes in 3D Collagen I culture: an in vitro physiological environment for the study of extracellular matrix and host cell interactions
Source: PeerJ. 2014 Apr 3;2:e317. doi: 10.7717/peerj.317 (PMC3994643; doi:10.7717/peerj.317)
Supplement: Table S1 — Viability was measured using a fluorescent Live/Dead assay (Invitrogen) and only samples with viability scores higher than 90% (green) were used to guarantee same viability as the control samples without PI mix treatment. Important to point out that as demonstrate in that table those viabilities are related to the combination effect of the PI mix in a 3D COL I (in RPMI media) context. [file peerj-02-317-s005.pdf]

| Protease inhibitors mix (PI mix) | | | | | | |
|---|---|---|---|---|---|---|
| **E-64** | **O-phenantroline** | **EDTA** | **Marimastat** | **Cistatin** | **AEBSF** | **Viability** |
| 10 µM | x | x | 120nM | 20ng/ml | 1mM | >90% |
| 20 µM | x | x | 120nM | 20ng/ml | 1mM | >90% |
| 20 µM | x | 500mM | 120nM | 20ng/ml | 1mM | No COL I polymerization |
| **100 µM** | **x** | **x** | **200nM** | **20ng/ml** | **1mM** | **>90%** |
| 200 µM | x | x | 300nM | 20ng/ml | 1mM | <90% |
| 100 µM | 5mM | x | 200nM | 20ng/ml | 1mM | <80% |
| 100 µM | 1mM | x | 200nM | 20ng/ml | 1mM | <80% |

Supplemental material: ***Leishmania amazonensis*** **3D COL I culture viability after PI mix treatment.** Viability was measured using a fluorescent Live/Dead assay (Invitrogen) and only samples with viability scores higher than 90% (green) were used to guarantee the same viability as the control samples without PI mix treatment. It is important to point out that, as demonstrated in the table, viabilities are related to the combinatorial effect of the PI mix in a 3D COL I (in RPMI media) context.
